# A fluorogenic biotracer for reliable detection of staphylococcal beta-lactamase: towards improved precision therapy in severe MSSA infections

**DOI:** 10.1093/jac/dkag268

**Published:** 2026-08-14

**Authors:** Céline Dupieux, Patricia Martins Simoes, Benjamin Youenou, François Vandenesch, Anne Tristan, Frédéric Laurent

**Affiliations:** French National Reference Centre for Staphylococci, Institut des Agents Infectieux, Hospices Civils de Lyon, Lyon, France; Centre for Integrative Research in Infectious Diseases and Immunology, Inserm U1111, CNRS UMR5308, ENS de Lyon, Université Lyon 1, Lyon, France; French National Reference Centre for Staphylococci, Institut des Agents Infectieux, Hospices Civils de Lyon, Lyon, France; Centre for Integrative Research in Infectious Diseases and Immunology, Inserm U1111, CNRS UMR5308, ENS de Lyon, Université Lyon 1, Lyon, France; French National Reference Centre for Staphylococci, Institut des Agents Infectieux, Hospices Civils de Lyon, Lyon, France; Centre for Integrative Research in Infectious Diseases and Immunology, Inserm U1111, CNRS UMR5308, ENS de Lyon, Université Lyon 1, Lyon, France; French National Reference Centre for Staphylococci, Institut des Agents Infectieux, Hospices Civils de Lyon, Lyon, France; Centre for Integrative Research in Infectious Diseases and Immunology, Inserm U1111, CNRS UMR5308, ENS de Lyon, Université Lyon 1, Lyon, France; French National Reference Centre for Staphylococci, Institut des Agents Infectieux, Hospices Civils de Lyon, Lyon, France; Centre for Integrative Research in Infectious Diseases and Immunology, Inserm U1111, CNRS UMR5308, ENS de Lyon, Université Lyon 1, Lyon, France; French National Reference Centre for Staphylococci, Institut des Agents Infectieux, Hospices Civils de Lyon, Lyon, France; Centre for Integrative Research in Infectious Diseases and Immunology, Inserm U1111, CNRS UMR5308, ENS de Lyon, Université Lyon 1, Lyon, France

## Abstract

**Background and objectives:**

Accurate detection of staphylococcal beta-lactamase remains a diagnostic challenge, despite its importance for guiding antibiotic therapy, particularly in severe infections, such as endocarditis and bacteraemia. Currently, no available method reliably detects beta-lactamase activity in all staphylococcal species. Here, we evaluated a novel fluorogenic biotracer, 352600-C (MOLSID, Lyon, France), designed to detect beta-lactamase activity in staphylococci.

**Methods:**

The 352600-C biotracer was incorporated into Mueller–Hinton agar plates onto which staphylococcal strains were subcultured at 37°C. Colony fluorescence was assessed at 4, 6 and 24 h using a 365 nm UV lamp. The assay was performed on a large collection of *bla*Z-positive (*n* = 124, including BlaZ types A, B, C and D) and *bla*Z-negative (*n* = 69) staphylococci from 18 species (*Staphylococcus aureus*, *n* = 100; *S.* non-*aureus*, *n* = 93). Molecular detection of the *bla*Z gene using WGS served as the gold standard. Phenotypic susceptibility to penicillins was also tested using EUCAST recommendations.

**Results:**

After 4, 6 and 24 h of incubation, the assay showed sensitivities of 92.2%, 96.9% and 100% for *S. aureus* and 50.0%, 88.3% and 100% for non-*aureus* staphylococci, respectively. None of the 69 *bla*Z-negative strains produced fluorescent colonies (specificity 100%). Notably, the assay detected all BlaZ types. Penicillin disc diffusion showed 98.4% sensitivity and 77.8% specificity for MSSA.

**Conclusions:**

This novel assay for the detection of staphylococcal beta-lactamase demonstrated simplicity, rapidity and excellent accuracy after 24 h incubation (100% sensitivity and specificity) across a genetically diverse and representative collection of clinical isolates, making it a valuable tool for routine diagnostics and antimicrobial stewardship.

## Introduction

With the rise of antimicrobial resistance, precision in antimicrobial prescribing has become more critical than ever. In severe infections caused by *Staphylococcus aureus*, such as bacteraemia and infective endocarditis (IE), non-optimal initial antimicrobial chemotherapy has a significant impact on mortality,^1–3^ highlighting the need for accurate detection of resistance mechanisms, particularly against beta-lactams. Although penicillin G and penicillin A are the most active beta-lactams against MSSA, their clinical use is often limited by the difficulty of reliably confirming the absence of beta-lactamase (the *bla*Z gene product). Interestingly, studies conducted between 2014 and 2023 have shown that the prevalence of *bla*Z-positive MSSA isolates varies between 56.9% and 80% across European countries.^4–7^ Moreover, recent data from the SNAP trial demonstrated the efficacy and interest of penicillin G (compared to anti-staphylococcal penicillins) in bacteraemia due to *bla*Z-negative MSSA isolates,^8^ further emphasizing the need for robust beta-lactamase detection tools.

Phenotypic methods, such as the benzylpenicillin disc edge test and nitrocefin-based assays, remain observer dependent and lack sensitivity. Molecular detection (mostly PCR), although accurate, is not routinely available in most clinical laboratories. To bridge this gap, we evaluated a novel fluorogenic biotracer (352600-C) developed by MOLSID SAS (Lyon, France), designed to detect staphylococcal beta-lactamase activity in an easily interpretable format compatible with routine microbiological workflows.^9^ In this study, we present the first clinical evaluation of this biotracer in a large and diverse collection of *S. aureus* and non*-aureus* staphylococcal isolates.

## Materials and methods

The 352600-C biotracer is a fluorogenic compound structurally derived from a beta-lactam core. While completely non-fluorescent in its initial conformation, upon hydrolysis by staphylococcal beta-lactamase, it produces an intensely green fluorescent precipitate that is detectable under UV light (365 nm). The biotracer was incorporated directly into Mueller–Hinton (MH) agar (10 µM), allowing detection of fluorescent colonies after streak inoculation and 24 h incubation at 37°C for staphylococcal beta-lactamase–positive (benzylpenicillin–resistant) isolates.

A panel of 193 well-characterized staphylococcal isolates was tested, including 100 *S. aureus* isolates (belonging to 60 sequence types and covering all BlaZ types) and 93 *S.* non-*aureus* (SNA) isolates belonging to all major human-associated species (*n* = 17). The presence of *bla*Z and BlaZ typing was determined using WGS for all strains (Supplementary data, available as Supplementary data at *JAC* Online). Colony fluorescence was assessed by two independent readers after 4, 6 and 24 h of incubation to evaluate signal kinetics. *S. aureus* ATCC25923 and *S. aureus* ATCC29213 were used as negative and positive controls, respectively, for each series of experiments. Particular attention was given to the distribution of BlaZ variants (A, B, C, D and others) and to the assay’s robustness across diverse phylogenetic backgrounds.

In comparison, phenotypic susceptibility testing was performed using 2026 EUCAST recommendations for isolates belonging to *S. aureus*, *Staphylococcus lugdunensis* and *Staphylococcus saprophyticus*,^10^ using a 1 unit benzylpenicillin (for *S. aureus* and *S. lugdunensis*) or a 2 µg ampicillin disc (for *S. saprophyticus*) onto MH agar (discs from i2a, Montpellier, France; MH agar from Bio-Rad, Marnes-la-Coquette, France).

## Results and discussion

Among the 124 *blaZ*-positive isolates, all yielded fluorescent colonies within 24 h (sensitivity 100%) (Table 1; Table S1). No false positives were observed among the 69 *bla*Z-negative strains (specificity 100%). Early fluorescence detection was observed in the majority of isolates: 92.7% (115/124) of *bla*Z-positive isolates fluoresced within 6 h (62/64, 96.9% for *S. aureus* and 53/60, 88.3% for SNA). Only 9/124 isolates (7.3%) required a full 24 h incubation to be adequately classified as positive. The assay performed uniformly across *bla*Z genotypes, with no signal discrepancies between BlaZ variants. Importantly, the assay maintained performance across *S. aureus* phylogenetic diversity (clonal complexes 1, 5, 8, 22, 30, etc.), and a large panel of SNA species (including *Staphylococcus epidermidis*, *S. lugdunensis*, *Staphylococcus hominis*, *Staphylococcus haemolyticus*, *Staphylococcus capitis*, *S. saprophyticus*).

**Table 1.** Sensitivity of the 352600-C biotracer (MOLSID, Lyon) for detecting staphylococcal penicillinase in a collection of 193 strains

	Positive strains, *n* (%)		
	4 h incubation	6 h incubation	24 h incubation
*S. aureus bla*Z*+* (*n* = 64)	59 (92.2)	62 (96.9)	64 (100)
*S. aureus bla*Z*−* (*n* = 36)	0	0	0
SNA *bla*Z*+* (*n* = 60)	30 (50.0)	53 (88.3)	60 (100)
SNA *bla*Z*−* (*n* = 33)	0	0	0
Total	89 (71.8)	115 (92.7)	124 (100)
BlaZ type A (*n* = 40)	32 (80.0)	37 (92.5)	40 (100)
BlaZ type B (*n* = 43)	21 (48.8)	39 (90.7)	43 (100)
BlaZ type C (*n* = 28)	24 (85.7)	27 (96.4)	28 (100)
BlaZ type D (*n* = 9)	9 (100)	9 (100)	9 (100)
Other BlaZ (*n* = 4)	3 (75.0)	3 (75.0)	4 (100)

*bla*Z, gene encoding staphylococcal beta-lactamase; SNA, *Staphylococcus* non-*aureus* (*S. caeli*, *n* = 1; *S. capitis*, *n* = 13; *S. caprae*, *n* = 1, *S. cohnii*, *n* = 2; *S. epidermidis*, *n* = 43; *S. haemolyticus*, *n* = 8; *S. hominis*, *n* = 3; *S. lugdunensis*, *n* = 5; *S. pasteuri*, *n* = 1; *S. pettenkoferi*, *n* = 6; *S. pseudintermedius*, *n* = 1; *S. pseudoxylosus*, *n* = 1; *S. saprophyticus*, *n* = 5; *S. sciuri*, *n* = 1; *S. simulans*, *n* = 1; *S. warneri*, *n* = 1).

Regarding the conventional testing for penicillinase according to EUCAST recommendations, 63/64 (98.4%) *bla*Z+ MSSA isolates were phenotypically resistant to benzylpenicillin, and 28/36 *bla*Z- MSSA isolates were phenotypically susceptible, leading to a low specificity of 77.8%. The five and four methicillin-susceptible isolates belonging to *S. saprophyticus* and *S. lugdunensis*, respectively, were well classified using ampicillin and benzylpenicillin discs, as indicated by EUCAST.

Antimicrobial susceptibility testing (AST) is essential for guiding early appropriate personalized therapy, supporting antimicrobial stewardship and informing infection prevention and control in hospitals. However, phenotypic AST lacks sensitivity for some specific resistance mechanisms, such as the detection of beta-lactamase production in staphylococci. The fluorogenic assay developed by MOLSID overcomes the limitations of current phenotypic methods for beta-lactamase detection. It provides an easy-to-interpret readout with a broad-spectrum applicability across staphylococci (both *S. aureus* and coagulase-negative staphylococci). The method is cost-effective, equipment-free and perfectly adapted to routine laboratory workflows. Compared with chromogenic cephalosporin tests (e.g. nitrocefin) or the benzylpenicillin disc diffusion assay, the 352600-C biotracer provides easier-to-read results and 100% sensitivity and specificity. In light of the SNAP trial data, which showed improved outcomes and less side effects for bacteraemia caused by *bla*Z-negative MSSA treated with penicillin G, this tool could enable an earlier adapted personalized therapy, reducing unnecessary exposure to broader-spectrum agents and making it of particular interest for antibiotic stewardship measures.

In summary, the 352600-C fluorogenic biotracer represents a significant advance in the detection of staphylococcal beta-lactamase. It is easy to implement, highly accurate and applicable to both methicillin-susceptible *S. aureus* and SNA. Its performance on a genetically diverse strain panel confirms its robustness. A commercial launch of ResiScreen Staph^TM^ (MOLSID) is planned for the first quarter of 2026, paving the way for more precise antimicrobial therapy in serious staphylococcal infections.

## Supplementary Material

dkag268_Supplementary_Data

## Supplementary data

Table S1 is available as Supplementary data at *JAC* Online.
