# Supplementary material for: A fluorogenic biotracer for reliable detection of staphylococcal beta-lactamase: towards improved precision therapy in severe MSSA infections
Source: J Antimicrob Chemother. 2026 Aug 14;81(9):dkag268. doi: 10.1093/jac/dkag268 (PMC13475114; doi:10.1093/jac/dkag268)
Supplement: Supplementary file 1 [file dkag268_supplementary_data.zip › JAC-2025-1832 supplementary 050626.docx]

# SUPPLEMENTARY MATERIAL

**A fluorogenic biotracer for reliable detection of staphylococcal beta-lactamase: towards improved precision therapy in severe MSSA infections**

Céline Dupieux^1,2*^, Patricia Martins-Simoes^1,2^, Benjamin Youenou^1,2^, François Vandenesch^1,2^, Anne Tristan^1,2^, Frédéric Laurent^1,2^

¹ French National Reference Centre for Staphylococci, Institut des Agents Infectieux, Hospices Civils de Lyon, Lyon, France

^2^ Centre for Integrative Research in Infectious diseases and Immunology, Inserm U1111, CNRS UMR5308, ENS de Lyon, Université Lyon 1, Lyon, France

# SUPPLEMENTARY INFORMATION ON GENOMIC ANALYSES

**Whole genome sequencing (WGS)**

WGS was performed on all 193 isolates. Bacterial DNA was extracted using the Maxwell RSC system (Promega). Libraries were prepared with Illumina DNA Prep (Illumina, San Diego, CA, US), and sequencing performed using MiSeq (2 x 300 bp) or NextSeq (2 x 150bp) instruments (Illumina).

Assemblies were generated with SPAdes v3.14. The MLST of *S. aureus* and *S. epidermidis* isolates was performed using *mlst* (Seemann T, *mlst* ***Github****,* <https://github.com/tseemann/mlst>) and the PubMLST website (<https://pubmlst.org/>) developed by Keith Jolley. Resistance genes, including *bla*Z, were detected with ABRicate and ResFinder v4.0. The genomes were annotated with Bakta v1.10.4.^1^

**BlaZ typing**

The BlaZ beta-lactamase type was determined based on the amino acids at positions 128 and 216 encoded by the *bla*Z gene after the annotation using Bakta and using the FASTA amino acid files.^2^

**Nucleotide sequence accession number**

Sequencing reads from all isolates included in the present study have been deposited in European Nucleotide Archive (ENA) under the accession number PRJEB112900.

**Supplementary references**

1. Schwengers O, Jelonek L, Dieckmann MA, *et al*. Bakta: rapid and standardized annotation of bacterial genomes via alignment-free sequence identification. *Microb Genom* 2021; **7**: 000685.

2. Nannini EC, Stryjewski ME, Singh KV, *et al.* Inoculum effect with cefazolin among clinical isolates of methicillin-susceptible *Staphylococcus aureus* : Frequency and possible cause of cefazolin treatment failure. *Antimicrob Agents Chemother* 2009; **53**: 3437–41.
